# Curcumin Attenuates Zearalenone-Induced Reproductive Damage in Mice by Modulating the Gut Microbe–Testis Axis

**DOI:** 10.3390/foods14152703

**Published:** 2025-07-31

**Authors:** Bangwang Peng, Shuaiju Guo, Junlong Niu, Yongpeng Guo, Zhixiang Wang, Wei Zhang

**Affiliations:** College of Animal Science and Technology, Henan Agricultural University, Zhengzhou 450046, China; pengbangwang@stu.henau.edu.cn (B.P.); guoshiju@stu.henau.edu.cn (S.G.); niujunlong@stu.henau.edu.cn (J.N.); guoyp@henau.edu.cn (Y.G.)

**Keywords:** zearalenone, curcumin, gut microbe–testis axis, inflammation, sperm quality

## Abstract

Zearalenone (ZEN), a mycotoxin commonly found in cereal crops and foods, induces testicular damage and disrupts gut microbial composition. Curcumin (CUR), a bioactive compound derived from turmeric, is known to enhance intestinal microbial balance and exhibit anti-inflammatory properties. This study aimed to investigate the mechanism by which CUR alleviates ZEN-induced reductions in sperm quality through the modulation of the gut microbiota–testis axis. Forty-eight 6-week-old Balb/c male mice were randomly assigned to four treatment groups: control (CON), CUR (200 mg/kg body weight CUR), ZEN (40 mg/kg body weight ZEN), and ZEN + CUR (200 mg/kg CUR + 40 mg/kg ZEN). The degree of sperm damage was quantified by assessing both the survival rate and the morphological integrity of the spermatozoa. CUR was found to mitigate ZEN-induced reductions in the testosterone levels, testicular structural damage, and disrupted spermatogenesis. Exposure to ZEN markedly perturbed the gut microbiota, characterized by increased relative abundances of Prevotella and Bacteroides and a concomitant reduction in Lactobacillus. These alterations were accompanied by pronounced activation of the IL-17A–TNF-α signaling axis, as demonstrated by elevated transcriptional and translational expression of pathway-associated genes and proteins. Co-administration of CUR effectively reinstated microbial homeostasis and mitigated ZEN-induced IL-17A pathway activation. In conclusion, ZEN induces testicular inflammation and reduced sperm quality by lowering testosterone levels and disrupting gut microbial balance, which drives the testicular IL-17A signaling pathway. CUR alleviates ZEN-induced testicular inflammation and sperm quality reduction by restoring beneficial gut microbes and testosterone levels.

## 1. Introduction

Zearalenone (ZEN) is a widely distributed mycotoxin primarily produced by species within the *Fusarium genus*, particularly *Fusarium graminearum* and *Fusarium culmorum* [1]. These fungi proliferate and synthesize ZEN under favorable environmental conditions, thereby contaminating cereal grains. Grains exhibit heightened vulnerability to ZEN contamination when stored under conditions characterized by elevated moisture, moderate-to-high temperatures, and sufficient oxygen availability. This issue is prevalent globally, with European and Asian countries reporting notably high levels of contamination [2]. A survey indicated that among 782 samples of crops, including corn and wheat, 90.15% of the corn samples and 79.55% of the wheat samples tested positive for ZEN [3]. The toxicity of ZEN and its byproducts is substantial, representing a significant hazard to human and animal health. Including reproductive toxicity, ZEN is chemically similar to estrogen and binds to the estrogen receptors, leading to estrogen interference and damage to the reproductive system [4]. Furthermore, ZEN exhibits various toxic effects, including intestinal toxicity, immunotoxicity, hepatotoxicity, and nephrotoxicity [5,6,7]. In male mammals, the testis serves as the principal gonad, responsible for spermatogenesis and the production of androgenic hormones such as testosterone, which is vital to male reproductive health [8]. Spermatogenic epithelial cells, located within the seminiferous tubules of the testis, play a crucial role in transforming spermatogonia into mature spermatozoa through a complex physiological process [9,10]. Testosterone, produced by Leydig cells in the testis, is essential for spermatogenesis [11] and also significantly regulates muscle growth, fat distribution, red blood cell production, and cognitive functions, thereby contributing to overall health [12]. Research shows that disruptions in the gut microbiota can cause testicular abnormalities and reduce sperm production. Moreover, such disruptions can interfere with spermatogenesis, ultimately leading to a non-reproductive state of the testicles [13]. Studies indicate that the gut microbiota can affect androgen metabolism and protection, further influencing spermatogenesis [14]. Improvement in the gut microbiota has been shown to enhance reproductive health [15]. Notably, fecal microbiota transplantation has been demonstrated to improve semen quality and spermatogenesis. Recent evidence has emerged demonstrating that exposure to ZEN induces structural abnormalities in the testes of mice and is associated with a downregulation of hormone levels [16]. Prolonged ZEN intake is associated with a reduction in Firmicutes/Bacteroidota and a decrease in the abundance of beneficial *Lactobacillus*, leading to microbial alterations in the intestinal tract of laying hens and subsequent intestinal damage [17]. Additionally, exposure to ZEN has been shown to affect the gut microbiota and follicular development in offspring during lactation [18]. Furthermore, ZEN treatment induces significant shifts in gut microbiota composition, characterized by a decrease in beneficial genera such as *Akkermansia* and *Ruminococcaceae*, and an increase in pathogenic *Staphylococcus aureus* [19]. Studies reveal that ZEN boosts the presence of certain bacteria like *Parabacteroides*, *Bacteroides*, and *Lachnospiraceae* depending on the dose, while it suppresses the glycerophospholipid metabolic pathway in bacteria [20].

Curcumin (CUR), extracted from the rhizomes of turmeric (*Curcuma longa* L.), falls into the category of polyphenols [21]. CUR exhibits a reddish-brown hue in alkaline conditions and turns yellow in neutral to acidic environments. It does not dissolve in water or ether but is soluble in ethanol, propylene glycol, glacial acetic acid, and alkaline solutions [22]. Turmeric has long been recognized in traditional medicine for its anti-inflammatory, anticancer, and digestive health properties. Curcumin, which constitutes up to 5% of turmeric’s composition, has gained significant attention due to the therapeutic benefits attributed to turmeric and the relative ease of curcumin extraction. According to the European Medicines Agency report, the NOEL for CUR is 250–320 mg/kg bw/day. In addition, previous studies have reported no discernible adverse effects following the sole administration of 200 mg/kg CUR to mice for 38 consecutive days [23]. Research indicates that Cur may offer several potential health benefits, including anti-inflammatory [24] and anticancer properties [25]. Zhang et al. [26] reported that CUR could mitigate dextran sulfate sodium salt-induced anxiety-like behaviors by modulating specific intestinal flora, highlighting its significant role in maintaining intestinal microbiota homeostasis. Furthermore, Tsao reported that CUR could alleviate reproductive dysfunction associated with metabolic disorders and improve sperm production defects induced by a high-fat diet [27]. Therefore, this study aimed to investigate whether CUR mitigates ZEN-induced testicular inflammation via the gut–testis axis and consequently alleviates ZEN’s detrimental effects on sperm quality by administering ZEN and CUR to mice via gavage.

## 2. Materials and Methods

The animal experiment protocol for this study was approved by the Animal Ethics Committee of Henan Agricultural University (HNND2023031434). All mice were cared for humanely in accordance with the standards outlined in the Guide for the Care and Use of Laboratory Animals [28].

### 2.1. Reagents

Forty-eight male Balb/c mice weighing 20–22 g were procured from Liaoning Changsheng Biotechnology Co., Ltd. (Benxi, China). CUR (purity ≥ 98%) was purchased from Dalian Meilun Biotechnology Co., Ltd. (Dalian, China). ZEN (purity ≥ 98%) was purchased from Shanghai Yuanye Biotechnology Center (Shanghai, China). A testosterone ELISA kit was obtained from Wuhan Pinofil Biotechnology Co., (Wuhan, China). Sperm rapid staining solution was sourced from Nanjing Jiancheng Technology Co., (Nanjing, China). Real-time PCR kits were sourced from Takara Biomedical Technology Co., (Dalian, China). for our experiments. Primers were designed using the NCBI database and synthesized by Sangon Biotech (Shanghai, China) [29]. Protease inhibitors were obtained from Solepol Biotech (Beijing, China). Three-color pre-staining markers (WJ103), TBS/Tween buffer (PS103s), electrophoresis buffer (PS105S), and transmembrane buffer (PS109S) were purchased from Yase Biomedical Technology Co., (Shanghai, China). The primary antibodies employed in protein immunoblotting for this assay included rabbit IL-17A, rabbit anti-TRAF6, rabbit anti-IL17RA, rabbit anti-TNF-α, and mouse anti-β-actin.

### 2.2. Treatment

After a week of acclimatization, forty-eight six-week-old mice were randomly assigned to one of four groups: a control (CON), a CUR-only group (CUR, 200 mg/kg b. w.), a ZEN-only group (ZEN, 40 mg/kg b. w.), and a group receiving both ZEN and CUR (200 mg/kg b. w. CUR + 40 mg/kg b. w. ZEN). The composition of the basic diet is outlined in Table S1. Olive oil was utilized as the solvent for both CUR and ZEN. To ensure uniformity, a consistent concentration of dimethyl sulfoxide (DMSO) was maintained throughout all experimental protocols, given ZEN’s solubility in DMSO.

Every day at 8:30, groups CON and ZEN were gavaged with 0.2 mL of olive oil, while groups CUR and ZEN + CUR received the equivalent volume of CUR in olive oil. Furthermore, at 11:30, the CON and CUR groups were administered 0.2 mL of olive oil via gavage, while the ZEN and ZEN + CUR groups received ZEN at the specified dosage through gavage. The body weights and behavioral modifications of the mice were recorded weekly. On day 29 of the study, the final body weights of the mice were measured. Following this, the animals were anesthetized with ether, and blood was collected from the ophthalmic vein. The blood samples were centrifuged at room temperature (3000 rpm for 10 min at 4 °C) to isolate the supernatant, which was then stored at −80 °C in a deep freezer. Furthermore, the unilateral testes of six mice from each treatment group were fixed in 4% paraformaldehyde, whereas the unilateral epididymis of three mice from each group was selected for the evaluation of sperm parameters. Collected mouse testis and small intestine tissues were frozen at −80 °C for subsequent analysis of real-time quantitative PCR and Western blot.

### 2.3. Testicular Index

The testes were excised and meticulously cleaned to remove adjacent adipose tissue, then weighed using an electronic balance. The testicular index was calculated using the following formula: (testicular mass in milligrams/body weight in grams) × 100% [30].

### 2.4. Testosterone Levels in the Serum

Blood was collected from the eyeballs to prepare serum, and serum testosterone levels in mice were assessed in each group following the guidelines provided by the ELISA detection kit [31].

### 2.5. Histopathological Assessment

Following fixation in 4% paraformaldehyde, the testes of the mice were embedded in paraffin. Sections of the specimens, each 5 μm thick, were prepared with a microtome and later stained with hematoxylin and eosin for histological analysis. Sections stained with hematoxylin and eosin were observed and documented using an Olympus microscope from Tokyo, Japan. Images were captured at magnifications of 200× and 400×. Sertoli cells, germ cells, and seminiferous tubules were examined, and the extent of testicular injury was evaluated [31].

### 2.6. Sperm Parameters Evaluation

Freshly collected epididymis samples were promptly transferred to an ultra-clean bench. Following the removal of external fatty tissue, the samples were finely minced and placed into a 2 mL centrifuge tube. To facilitate sperm release, 1 mL of saline was added. The tube was subsequently incubated in a 37 °C water bath for 15 min to prepare a fresh sperm suspension. A small aliquot of this suspension was transferred onto a preheated hematocrit slide, where the count of non-motile spermatozoa within five designated squares was recorded as ‘B’, whereas the total spermatozoa count was noted as ‘A’. Sperm viability was then calculated as a percentage using the following formula: (A − B)/A.

For the assessment of sperm morphology, a 10:1 dilution was prepared by mixing 20 μL of the sperm suspension from each mouse with 180 μL of saline solution. A 10 μL aliquot of this diluted sample was spread onto a glass slide, air-dried for 5 min, and then fixed using a 4% formaldehyde solution for 15 min. Following preparation, the slides were stained with a 1% eosin solution for a duration of 30 min. Subsequently, they were examined and photographed using an optical microscope [32].

### 2.7. Real-Time Quantitative PCR Analysis

Total RNA was extracted from murine testicular tissue employing TRIzol reagent as per the manufacturer’s protocol. The concentration of the RNA was quantified by measuring optical density at 260/280 nm with a NanoDrop One/OneC spectrophotometer (Model P800, Implen, Munich, Germany). Total RNA was converted into cDNA through reverse transcription using a designated RNA reverse transcription kit. Primers, detailed in Table S2 and synthesized by Sangon Biotech (Shanghai, China), were employed in this procedure. Relative expression levels were calculated using the 2^−ΔΔCt^ technique, with amplification of cDNA performed. β-actin was utilized as an internal control for normalization [33].

### 2.8. Western Blot Analysis

Total proteins were extracted from testicular tissue by complete lysis with lysis buffer, followed by measurement of the protein concentration using the Total Protein Assay Kit in accordance with the provided instructions (Shanghai, China). After extraction, proteins were mixed with Protein Sampling Buffer and then boiled for 10 min. After electrophoresis and subsequent membrane transfer, the protein samples were blocked with 5% skim milk for 2 h, incubated with the primary antibody at 4 °C overnight, and then with the secondary antibody at room temperature for 1 h. After the Western blot development process, the target bands’ grayscale intensities were assessed using ImageJ software (V6.0; National Institutes of Health, Bethesda, MD, USA) [34].

### 2.9. Gut Microbiota Analysis

Referring to the method described by Teng [35], DNA was extracted from the contents of the cecum using a specialized kit. This was followed by the amplification of the V3-V4 segment of the 16S rRNA gene utilizing the primers 338F: ACTCCTACGGGGGAGGCAGCA and 806R: GGACTACHVGGTWTCTAAT. DNA library preparation and sequencing of the 16S rRNA gene, along with data analysis services, were provided by Personal Technology Co., (Shanghai, China). The α-diversity measures were calculated in QIIME using OTU data, β-diversity was visualized through Principal Coordinates Analysis (PCoA), and the species composition was examined.

### 2.10. Statistical Analysis

Statistical analyses were performed using SPSS 25.0 software. All data are presented as the mean ± SEM. For comparisons between two groups, a *t*-test was employed. Data from more than two groups were analyzed via analysis of variance (ANOVA), with Tukey’s correction applied for multiple comparisons. Histograms were generated using GraphPad Prism 7, with values within a row that have different superscripts (a, b, c) indicating significant differences (*p* < 0.05).

## 3. Results

### 3.1. Effects of ZEN and CUR on Testicular Injury in Mice

**Figure 1:**
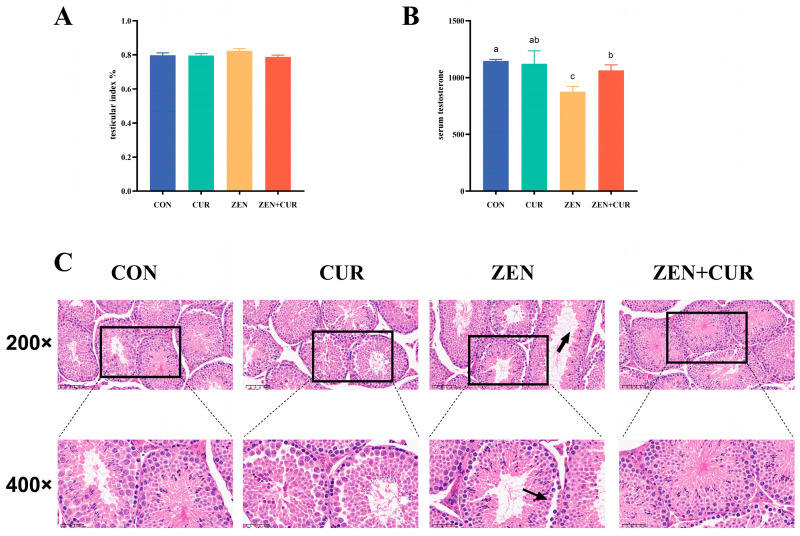
Effects of ZEN and CUR on mouse testis. (**A**) Testis index. (**B**) Serum testosterone. (**C**) Tissue sections of testis (n = 6), black arrow indicates cell vacuolation. Results are presented as mean ± SEM, with values within a row that have different superscripts (a, b, c) indicating significant differences (*p* < 0.05).

As shown in Figure 1A, there was no significant change in the testicular index in any of the treatment groups (*p* > 0.05). The serum testosterone levels between the CUR and CON groups were not significantly different (*p* > 0.05). The addition of ZEN significantly decreased the serum testosterone levels (*p* < 0.05), whereas CUR supplementation markedly restored the serum testosterone levels (*p* < 0.05) (Figure 1B). The testicular tissue in the CON and CUR groups appeared morphologically normal, with tightly aligned spermatogonial tubules. In contrast, the presence of ZEN resulted in cellular detachment, and the configuration of spermatogenic tubules became disorganized and lax. However, the spermatogonial tubules in the ZEN + CUR group were tightly arranged and showed a recovery to levels comparable to the CON group (Figure 1C).

### 3.2. ZEN and CUR on Sperm Survival in Mice

**Figure 2:**
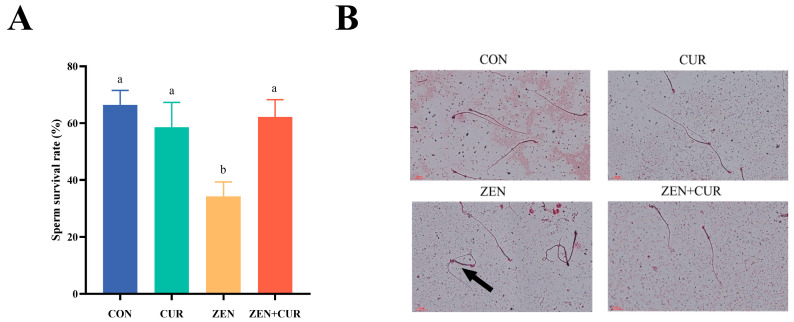
ZEN and CUR on sperm survival in mice (n = 6). (**A**) Sperm survival rate. (**B**) Sperm morphology, black arrows indicate malformations of the sperm. Results are presented as mean ± SEM, with values within a row that have different superscripts (a, b) indicating significant differences (*p* < 0.05).

As shown in Figure 2A, ZEN exposure significantly reduced sperm survival to 34.25% (*p* < 0.05), whereas CUR supplementation restored it to 62.22% (*p* < 0.05). Normal sperm morphology was noted in both the CON and CUR groups. However, the addition of ZEN caused deformities in sperm morphology, which returned to normal following CUR supplementation (Figure 2B). These findings indicate that ZEN can induce sperm damage in mice, leading to deformities, and that CUR effectively mitigates this damage and morphological impairment.

### 3.3. Effects of ZEN and CUR on Mouse Sperm Synthesis Pathway Genes

**Figure 3:**
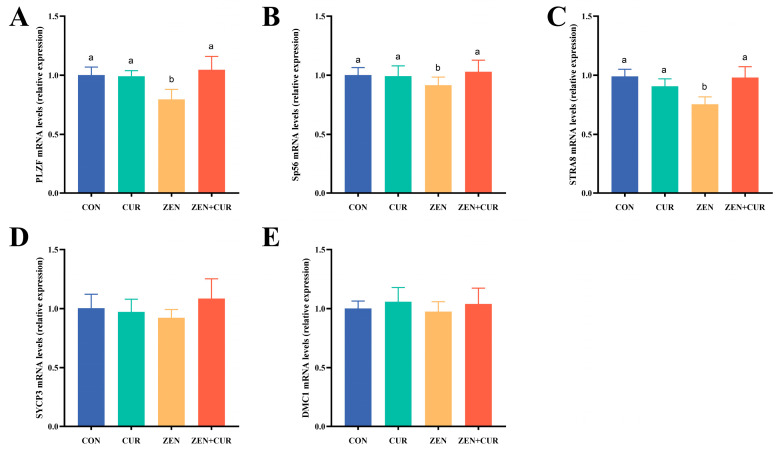
Effects of ZEN and CUR on mouse sperm synthesis pathway genes (n = 6). (**A**–**E**) The expression of sperm synthesis pathway genes. Results are presented as mean ± SEM. Values within a row that have different superscripts (a, b) indicate significant differences (*p* < 0.05).

As illustrated in Figure 3, the expression levels of the *SYCP3* and *DMC1* genes did not significantly differ between the treatment groups (*p* > 0.05). In the ZEN group, the expression levels of *PLZF*, *SP56*, and *STRA8* were significantly reduced compared to the CON group (*p* < 0.05). Conversely, the ZEN + CUR group exhibited significant upregulation of these genes when compared to the ZEN group (*p* < 0.05).

### 3.4. Expression of IL-17A Signaling Pathway Genes and Proteins

**Figure 4:**
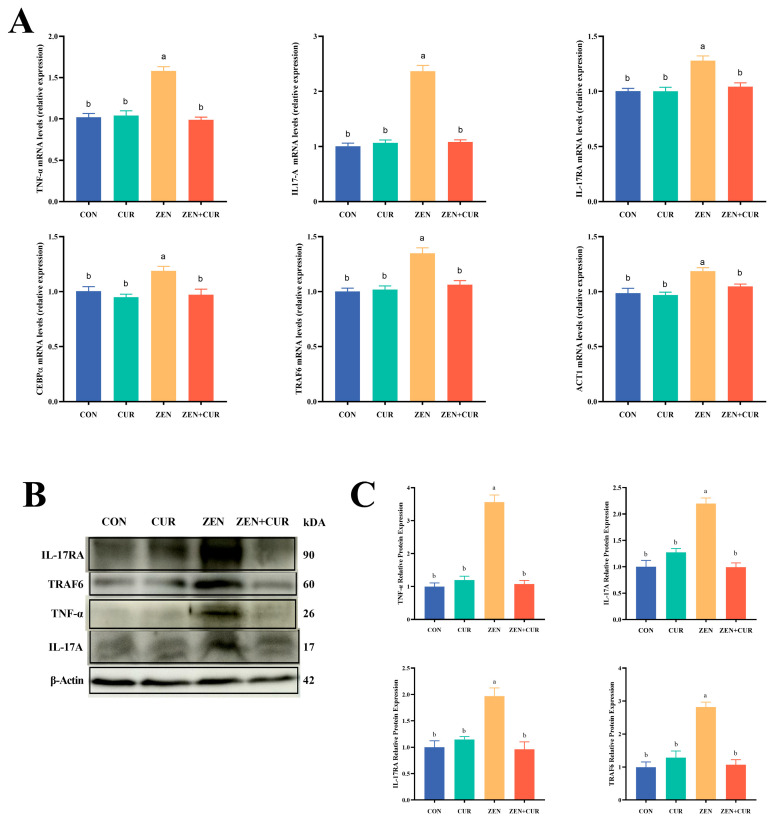
Expression of IL-17A signaling pathway genes and proteins. (**A**) Expression of IL-17A signaling pathway genes (n = 6). (**B**) ZEN induced increased levels of IL-17A signaling pathway protein expression in mouse testis. Each band shown is derived from 3 independent experiments. (**C**) The quantification of (**B**). Results are presented as mean ± SEM, with values within a row that have different superscripts (a, b) indicating significant differences (*p* < 0.05).

As shown in Figure 4A, the expression levels of *IL-17A*, *IL-17RA*, *TRAF6*, *CEBP-α*, *ACT1*, and *TNF-α* were significantly elevated in the ZEN group compared to the CON group (*p* < 0.05). Conversely, the expression of *IL-17A*, *IL-17RA*, *TRAF6*, *CEBP-α*, *ACT1*, and *TNF-α* in the ZEN + CUR group was significantly reduced in comparison to the ZEN group (*p* < 0.05). Western blot analysis further confirmed that the relative expression levels of the TRAF6, IL-17RA, IL-17A, and TNF-α proteins were markedly higher in the ZEN group than in the CON group (*p* < 0.05). Also, the relative expression of TRAF6, IL-17RA, IL-17A, and TNF-α proteins decreased significantly in the ZEN + CUR group compared to the ZEN group (*p* < 0.05) (Figure 4C).

### 3.5. Effects of ZEN and CUR on the Gut Microbiota of Mice

**Figure 5:**
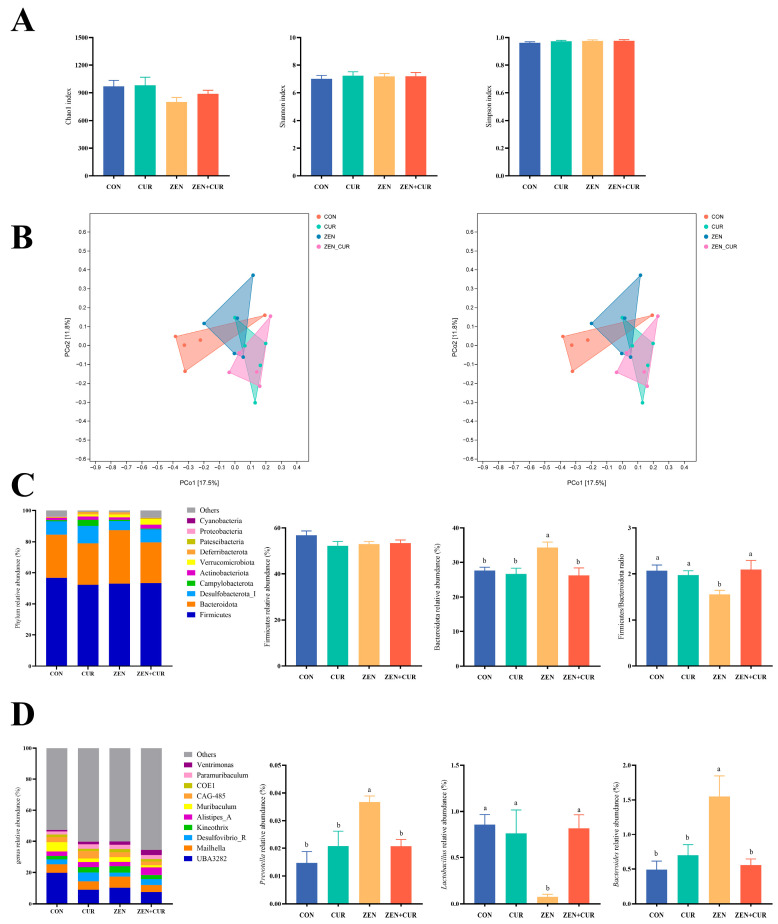
Effects of ZEN and CUR on the gut microbiota of mice (n = 5). (**A**) α-diversity. (**B**) β-diversity. (**C**) Phylum relative abundance, Firmicutes and Bacteroidota relative abundance, the ratio of Firmicutes/Bacteroidota. (**D**) Genus relative abundance, *Bacteroides*, *Prevotella*, and *Lactobacillus* relative abundance. Results are presented as mean ± SEM, with values within a row that have different superscripts (a, b) indicating significant differences (*p* < 0.05).

As shown in Figure 5A, there were no significant differences in the Chao1 index, Simpson index, and Shannon index among the treatment groups (*p* > 0.05), indicating that the addition of ZEN and CUR did not cause significant changes in α-diversity. PCoA and NMDS analyses indicated that the inclusion of ZEN resulted in distinct separation of gut microbial communities, signifying alterations in their β-diversity (Figure 5B). As shown in Figure 5C, Firmicutes and Bacteroidota were the predominant phyla in all treatment groups, with no significant difference in the relative abundance of Firmicutes among the groups (*p* > 0.05). The relative abundance of Bacteroidota was significantly higher in the ZEN group compared to the CON group (*p* < 0.05), whereas the ZEN + CUR group exhibited a significant decrease in Bacteroidota relative abundance compared to the ZEN group (*p* < 0.05). No significant variation in the relative abundance of Firmicutes was observed among the treatment groups (*p* > 0.05). The Firmicutes/Bacteroidota ratio was significantly lower in the ZEN group compared to the CON group (*p* < 0.05), whereas the ratio was significantly higher in the ZEN + CUR group compared to the ZEN group (*p* < 0.05). The top ten genera in terms of relative abundance are shown for each treatment group. The relative abundance of *Prevotella* and *Bacteroides* was significantly higher (*p* < 0.05) in the ZEN group compared to the CON group, while the relative abundance of *Prevotella* and *Bacteroides* was significantly lower (*p* < 0.05) in the ZEN + CUR group compared to the ZEN group. The relative abundance of *Lactobacillus* was significantly lower (*p* < 0.05) in the ZEN group compared to the CON group, whereas the relative abundance of *Lactobacillus* was significantly higher (*p* < 0.05) in the ZEN + CUR group compared to the ZEN group (Figure 5D).

### 3.6. Correlation Analysis Between Differential Bacteria and Sperm Synthesis Genes

**Figure 6:**
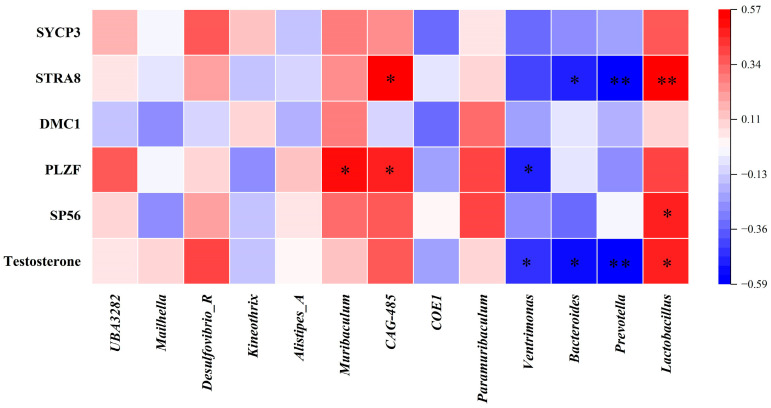
Correlation analysis between differential bacteria and sperm synthesis genes. * *p* < 0.05, ** *p* < 0.01.

As shown in Figure 6, Spearman correlation analysis was conducted to assess the associations between different bacteria and sperm synthesis genes. The results revealed that testosterone was negatively correlated with *Ventrimonas*, *Bacteroides*, and *Prevotella* while it was positively correlated with *Lactobacillus*. *SP56* was positively correlated with *Lactobacillus*. *PLZF* was positively correlated with *Muribaculum* and *CAG-485* while it was negatively correlated with *Ventrimonas*. *STRA8* was positively correlated with *CAG-485* and *Lactobacillus* while it was negatively correlated with *Bacteroides* and *Prevotella*.

## 4. Discussion

ZEN, a common mycotoxin, is prevalent in crops and foods. During storage, grains must be safeguarded against ZEN contamination under elevated temperature and relative humidity conditions [36]. Commonly employed strategies for the detoxification of ZEN encompass physical, chemical, and biological approaches [37]. Testosterone, a crucial hormone that influences libido and sexual function, is frequently utilized to assess reproductive health in male animals [38]. Low testosterone levels can result in decreased sperm production and diminished fertility. Due to its structural similarity to estradiol, ZEN can competitively bind to ER, thereby impacting testosterone levels. CUR is often used as a health promoter owing to its superior anti-inflammatory, antioxidant, and gut flora-regulating properties. In this study, ZEN markedly reduced the serum testosterone levels in mice, and this decline was reversed upon supplementation with CUR. Previous research has indicated that ZEN exposure significantly lowers the serum testosterone levels in rodents of all ages, leading to impaired reproductive performance and classifying ZEN as a potential endocrine disruptor [39]. These findings align with those of the current study. Tissue sections are routinely employed to identify and classify various diseases and injuries. In the present study, pathological examination revealed increased cellular vacuolization in the testicular morphology of the ZEN group, whereas CUR supplementation significantly reduced this cellular vacuolization to levels comparable to the control group. Furthermore, studies have demonstrated that ZEN exposure leads to testicular injury, which is characterized by disorganization of the seminiferous tubules, detachment of luminal cells, and increased apoptosis [40]. These results are consistent with our findings, indicating that ZEN is a detrimental substance that causes testicular damage, whereas CUR exhibits considerable potential in alleviating testicular injuries induced by ZEN.

In the animal organism, the gut microbiota plays a critical role in health maintenance, with the imbalance of this microbiota often being a significant contributing factor to various functional disorders [41]. Research indicates a strong connection between gut microbiota and the reproductive system in mammals, with changes in gut flora structure identified as a primary contributor to reproductive system disturbances [42]. Furthermore, microbial processes generate essential molecules that support key functions in nutrition, immunity, and hormonal balance, thereby benefiting the male reproductive system through the bloodstream [43]. ZEN exposure perturbs the intestinal microbiota of animals, manifesting as decreased α-diversity, diminished populations of beneficial taxa, and concomitant expansion of potentially pathogenic genera, ultimately compromising host health [44]. Gut microbial α-diversity reflects the abundance and uniformity of the flora; microorganisms demonstrating higher α-diversity tend to exhibit better adaptation to environmental changes and resistance to pathogens. Meanwhile, the analysis of gut microbial β-diversity highlights the differences in microbial communities across individuals. In our experiment, ZEN stimulation did not result in a decrease in α-diversity, but it did cause changes in β-diversity, which were subsequently reversed by CUR supplementation. ZEN stimulation significantly reduced the abundance of *Lactobacillus* and the ratio of Firmicutes/Bacteroidota whereas it increased the overall abundance of Bacteroidota and the abundance of *Prevotella*. Supplementation with CUR rectified these changes, restoring parity with the control group. It was reported that ZEN disrupted the microbiota composition in SD rats, leading to a notable decrease in beneficial bacterial genera such as *Akkermansia* and *Ruminococcaceae*, alongside a significant increase in pathogenic *staphylococci* [45]. Another study indicated that a dosage of 2.0 mg/kg ZEN significantly impacted the gut microbiota of 30-week-old hens, specifically decreasing the ratio and abundance of *Lactobacillus* in the intestinal thick-walled and anamorphic phyla, whereas it concurrently increased the abundance of anamorphic phyla, paraphyletic phyla, and desulfurizing Vibrio [17]. Additionally, studies have shown that CUR markedly enhances the population of beneficial microbes, such as *Bifidobacteria*, *Lactobacilli*, and bacteria that produce butyrate—a short-chain fatty acid beneficial for gut health—while also decreasing the levels of potentially harmful bacterial groups, such as *Prevotellaceae*, *Coriobacterales*, *Enterobacteria*, and *Rikenellaceae* [46]. Zhu also reported that Cur promotes an increase in beneficial bacteria and a corresponding decrease in harmful bacteria [47]. These findings are consistent with the results of the present study, indicating that ZEN causes disruption in the gut microbial structure whereas CUR effectively maintains the stability of the gut flora.

The IL-17A signaling pathway, which is essential for immune regulation, plays a critical role within the body [48,49], as its activation triggers the secretion of inflammatory factors such as TNF-α, IL-1β, and IL-6 [49,50]. The gut microbiota is recognized as a significant regulator of IL-17A production and function [51]. In this study, ZEN stimulation activated the IL-17A signaling pathway, resulting in increased expression levels of related genes and proteins, subsequently elevating TNF-α expression at both the mRNA and protein levels, a phenomenon that was reversed by CUR. Liu reported that glyphosate stimulation also activated the IL-17A signaling pathway and upregulated TNF-α mRNA expression in rat testes, which led to inflammation [32]. Similar findings were observed in a study where microplastic stimulation resulted in increased expression of IL-17A signaling-related genes, contributing to the upregulation of TNF-α mRNA expression and, ultimately, testicular dysfunction in mice [52]. These studies corroborate the findings of the present trial, suggesting that ZEN induces testicular inflammation by altering the gut microbial composition, which in turn activates the IL-17A signaling pathway. Furthermore, CUR has demonstrated potential to alleviate inflammation in the testis.

Sperm quality is a crucial factor in male reproductive health. A decline in sperm quality significantly contributes to reproductive dysfunction in male animals. The results of this experiment showed that the addition of ZEN reduced sperm survival to 34.25%, while the addition of CUR increased it to 62.22%, induced sperm deformities, and decreased the expression of genes involved in sperm synthesis. The addition of CUR effectively reversed these adverse effects. Prior studies have reported that ZEN stimulation resulted in decreased sperm concentration and viability, along with increased sperm malformations [53]. Additionally, it has been noted that CUR alleviates the reduction in sperm quality associated with a low-carbohydrate diet [27]. The findings from these studies correspond with those of the current experiment, showing that sperm quality is negatively affected by ZEN stimulation, whereas CUR is associated with beneficial improvements.

## 5. Conclusions

In summary, ZEN exposure causes testicular inflammation, which may lead to decreased sperm quality. This mechanism may involve a reduction in testosterone levels and a disruption of the gut microbiota composition. The disturbance of gut microbes drives the activation of the testicular IL-17A signaling pathway. Conversely, CUR appears to mitigate ZEN-induced testicular inflammation and reduced sperm quality by enhancing the abundance of beneficial gut microbes and testosterone levels.

## Data Availability

The original contributions presented in this study are included in the article/Supplementary Materials. Further inquiries can be directed to the corresponding authors.

## Supplementary Materials

The following supporting information can be downloaded at: https://www.mdpi.com/article/10.3390/foods14152703/s1, Table S1: Composition and nutrient levels of the basal diet for mice (air-dry basis); Table S2: Primer sequence of target and reference genes.

## Abbreviations

The following abbreviations are used in this manuscript: ZEN, zearalenone; CUR, curcumin; DMSO, dimethyl sulfoxide; PLZF, zinc finger and BTB domain containing 16; Sycp3, synaptonemal complex protein 3; DMC1, DNA meiotic recombinase 1; STRA8, stimulated by retinoic acid gene 8; SP56, zona pellucida 3 receptor; IL-17A, interleukin 17A; IL-17RA, interleukin 17 receptor A; TRAF6, TNF receptor-associated factor 6; Cebp-α, CCAAT/enhancer binding protein alpha; ACT1, TRAF3 interacting protein 2; TNF-α, tumor necrosis factor-α.
