# Supplementary material for: Curcumin Attenuates Zearalenone-Induced Reproductive Damage in Mice by Modulating the Gut Microbe–Testis Axis
Source: Foods. 2025 Jul 31;14(15):2703. doi: 10.3390/foods14152703 (PMC12345740; doi:10.3390/foods14152703)
Supplement: Supplementary file 1 [file foods-14-02703-s001.zip › foods-3750268-supplementary.pdf]

Table S1. Composition and nutrient levels of the basal diet for mice (air-dry basis).

Items (%)	Content
Corn	30.00
Wheat offal	17.00
Soybean Meal	23.20
Flour	23.00
Soybean oil	1.20
Dicalcium Phosphate	1.50
Fish Meal	1.00
Limestone Flour	1.60
Pre-mixed Feed (1%)[1]	1.00
Sodium Chloride	0.50
Total	100.00
Nutrient levels	
Gross Energy kcal/J	3.44
Crude Protein %	24.02
Crude Fat %	12.95
Carbohydrates %	63.03

[1]Provided per kg of diet: methionine + cysteine 8.00 g, lysine 13.90 g, tryptophan 2.50 g, arginine 12.00 g, leucine 17.60 g, isoleucine 10.50 g, threonine 8.80 g, valine 11.90 g, Histidine 5.60 g, phenylalanine + tyrosine 16.80 g; vitamin A 20, 000.00 IU, vitamin D 1667.00 IU, vitamin E 182.00 mg, vitamin K 8.00 mg, vitamin B1 20.23 mg, vitamin B2 20.00 mg, vitamin B6 15.00 mg, vitamin B12 0.03 mg, niacin 70.00 mg, pantothenic acid 25.00 mg, biotin 0.30 mg, choline 1250.00 mg, folic acid 10.00 mg; sodium 2.83 g, magnesium 2.77 g, potassium 8.20 g, copper 12.41 mg, iron 158.60 mg, manganese 88.10 mg, zinc 50.70 mg, selenium 0.20 mg, iodine 0.90 mg.

Table S2. Primer sequence of target and reference genes.

Gene	Accession number		Sequence (5'-3')	Product size (bp)
PLZF	NM_001033324.3	F	ATGCGACTGGCTGGGACTTTG	128
		R	TGCTGGCTGTTTCGGTGGAAG	
Sycp3	NM_011517.2	F	TGGTGCCTGGTGGAAGAAAGC	141
		R	CTGGAGCCTTTTCATCAGCAACATC	
DMC1	NM_001278226.1	F	TGGTGGACACATTCTGGCTCAC	82
		R	AATCTTGGCGATCCTCAGTTCTCC	
STRA8	NM_001410379.1	F	GAGGAAGGAGACGAAGAAGGAGAAG	139
		R	GCAACAGAGTGGAGGAGGAGTG	
SP56	NM_001420657.1	F	CGGTGTGAGGTCCAAGGTAAAGG	109
		R	TCCCACTGTGCTTCCCATTGC	
IL-17A	NM_010552.3	F	CTCAGACTACCTCAACCGTTCCAC	132
		R	TCCAGCTTTCCCTCCGCATTG	

IL-17RA	XM_006505618.2	F	GAGCCGACAGAAGCAGGAGATG	136
		R	CTTCCAGTGGTCACACCGTAGC	
TRAF6	NM_001303273.1	F	AGGAATCACTTGGCACGACACTTG	101
		R	TCGCACGGACGCAAAGCAAG	
Cebp-α	NM_001287514.1	F	TCGGTGGACAAGAACAGCAACG	140
		R	CGGTCATTGTCACTGGTCAACTCC	
ACT-1	NM_134000.3	F	CCAGAGCATCGGTTCACACTTACG	140
		R	CCACTGAAGGAGTCTGCCAAAGC	
TNF-α	NM_001278601.1	F	GATCGGTCCCCAAAGGGATG	118
		R	CCACTTGGTGGTTTGTGAGTG	
β-actin	NM_007393.5	F	CTACCTCATGAAGATCCTGACC	90
		R	CACAGCTTCTCTTTGATGTCAC	

[1]*PLZF* zinc finger and BTB domain containing 16, *Sycp3* synaptonemal complex protein 3, *DMC1* DNA meiotic recombinase 1, *STRA8* stimulated by retinoic acid gene 8, *SP56* zona pellucida 3 receptor, *IL-17A* interleukin 17A, *IL-17RA* interleukin 17 receptor A, *TRAF6* TNF receptor-associated factor 6, *Cebp-α* CCAAT/enhancer binding protein alpha, *ACT-1* TRAF3 interacting protein 2, *TNF-α* tumor necrosis factor.
